# Beyond logistic regression: structural equations modelling for binary variables and its application to investigating unobserved confounders

**DOI:** 10.1186/1471-2288-6-13

**Published:** 2006-03-15

**Authors:** Emil Kupek

**Affiliations:** 1 National Perinatal Epidemiology Unit, Institute of Health Sciences, University of Oxford, UK

## Abstract

**Background:**

Structural equation modelling (SEM) has been increasingly used in medical statistics for solving a system of related regression equations. However, a great obstacle for its wider use has been its difficulty in handling categorical variables within the framework of generalised linear models.

**Methods:**

A large data set with a known structure among two related outcomes and three independent variables was generated to investigate the use of Yule's transformation of odds ratio (OR) into Q-metric by (OR-1)/(OR+1) to approximate Pearson's correlation coefficients between binary variables whose covariance structure can be further analysed by SEM. Percent of correctly classified events and non-events was compared with the classification obtained by logistic regression. The performance of SEM based on Q-metric was also checked on a small (N = 100) random sample of the data generated and on a real data set.

**Results:**

SEM successfully recovered the generated model structure. SEM of real data suggested a significant influence of a latent confounding variable which would have not been detectable by standard logistic regression. SEM classification performance was broadly similar to that of the logistic regression.

**Conclusion:**

The analysis of binary data can be greatly enhanced by Yule's transformation of odds ratios into estimated correlation matrix that can be further analysed by SEM. The interpretation of results is aided by expressing them as odds ratios which are the most frequently used measure of effect in medical statistics.

## Background

### Statistical problems that require going beyond standard logistic regression

Although logistic regression has become the cornerstone of modelling categorical outcomes in medical statistics, separate regression analysis for each outcome of interest is hardly challenged as a pragmatic approach even in the situations when the outcomes are naturally related. This is common in process evaluation where the same variable can be an outcome at one point in time and a predictor of another outcome in future. For example, preterm delivery is both an important obstetric outcome and a risk factor for low birthweight, which in turn can adversely affect future health. Sequential nature of these outcomes is not encompassed by repeated measures models which deal with the same outcome at different time points. Another example of a research problem difficult to handle by logistic regression model is when an outcome is determined not only by direct influences of the predictor variables but also by their unobserved common cause. For example, survival time since the onset of an immune system disease may be adversely affected by concomitant occurrence of various markers of disease progression indicating immunosupression as an underlying common factor, the latter being an unobserved latent variable whose estimation requires solving a system of related regression equations.

Structural equation modelling (SEM) is a very general statistical framework for dealing with above issues. In recent years, it has been increasingly used in medical statistics. In addition to traditional areas such as psychometric properties of health questionnaires and tests, behavioural genetics [1], measurement errors [2] and covariance structure in mixed regression models [3] have received particular attention. In addition to specific applications, important research methodology issues in SEM have been given more space in medical statistics, among which a comparison with multiple regression [4], the relevance of latent variable means in clinical trials [5] and power of statistical tests [6] deserve special attention.

However, a great obstacle for wider use of SEM has been its difficulty in handling categorical variables. The aim of this paper is to briefly review main aspects of this difficulty and to demonstrate a new approach to this problem based on a simple transformation. Two examples with both simulated and real data are provided to illustrate this approach.

**Figure 1:**
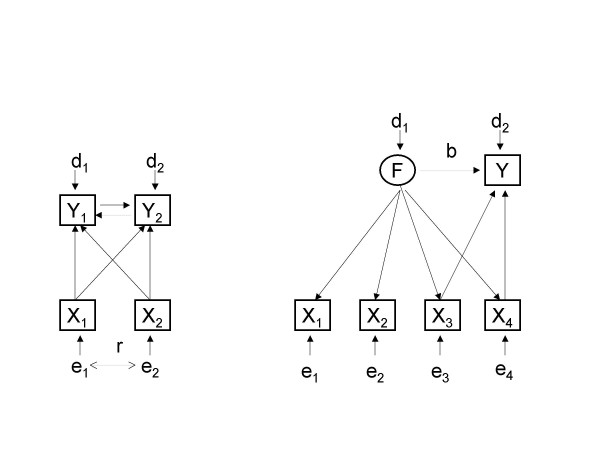
Statistical problems needing SEM approach.

SEM includes both observed and unobserved (latent) variables such as common factors and measurement errors. The Linear Structural Relationships (LISREL) model [7] was the first to spread in psychometric applications due to the availability of software. Other formulations of SEM and corresponding software emerged (see [8] for an overview). The details of these models, as well as important issues regarding their identifiability, estimation and robustness, are beyond the scope of this work but an illustration of the situations where SEM is needed is presented instead (Figure 1). As a general rule, SEM is indicated when more than one regression equation is necessary for statistical modelling of the phenomena under investigation.

The left part of Figure 1 shows a situation where two outcomes, denoted *Y*_1 _and *Y*_2_, are mutually related (a feed-back loop) and influenced by two predictors, denoted *X*_1 _and *X*_2_. For example, the outcomes could be demand and supply of a particular health service or risk perception and incidence of a particular health problem. The predictor variables' error terms, denoted *e*_1 _and *e*_2_, may be correlated (*r*) if an important variable influencing both predictors is omitted, i.e. in the case of bias in exposure measures. The terms *d*_1 _and *d*_2 _indicate disturbances of the two outcomes. The right part of Figure 1 illustrates a combination of common factors and regression model. In this case, it is of interest to test whether the outcome *Y *is determined not only by direct influences of the predictor variables, denoted *X*_1_, *X*_2_, *X*_3 _and *X*_4_, but also by their latent determinant as indicated by the regression coefficient *b*.

SEM has received many criticisms, most of which have been concerned with vulnerability of complex models relying on many assumptions, as well as with uncritical use and interpretation of SEM. These are well placed concerns but are not intrinsic to SEM; even well known and widely applied techniques such as regression share the same concerns. Complex phenomena require complex models whose inferential aspects are more prone to error as the number of parameters increases. SEM is often the only statistical framework by which many of these issues can be addressed by testing and comparing the models obtained [9].

### Handling categorical variables in SEM

Specific criticism regarding the treatment of categorical and ordinal variables in SEM has been a strong deterrent for its wider use. Naive treatment of binary and ordered categorical variables as if they were normally distributed in some SEM applications was partly due to the lack of viable alternatives in its early days. Inadequate use of standardized regression coefficients as the measures of effect in some SEM applications was also criticised [10]. Even when distributional properties of categorical variables were taken into account, the interpretation of SEM parameter estimates in terms of impact measures such as attributable risk was not applied. Standard errors and confidence limits – rarely used in SEM – are generally underestimating structural model uncertainties such as selection of relevant variables and correct specification of their influences.

A recent review of handling categorical and other non-normal variables in SEM [11] listed four main strategies: a) asymptotic distribution free (ADF) estimators adjusting for non-normality by taking into account kurtosis in joint multivariate distribution [12], b) the use of robust maximum likelihood estimation or resampling techniques such as jacknife or bootstrap to obtain the standard errors of SEM parameters as these are most affected by departure from multivariate normality [13], c) calculating polyserial, tetrachoric or polychoric correlations for pairs of variables with non-normal joint distribution by assuming that these have an underlying (latent) continuous scale whose large sample joint distribution is bivariate normal, then using these correlations as the input for SEM [14], and d) estimating probit or logit model scores for observed categorical variables as the first level, then proceeding with SEM based on these scores as the second-level [15]. The ADF estimation generally requires large samples to keep the type II error at a reasonable level and extremely non-normal variables such as binary may be difficult to handle with sufficient precision. The last two strategies critically depend on how well the first-level model fits the data.

A review of statistical models for categorical data reveals the lack of a method capable of handling more than one regression equation [16]. Although log-linear models for contingency tables may analyse related categorical outcomes and their relationship with independent variables, possibly complex interactions between the variables in the model do not indicate the direction of influences as in regression models. This underlines the need for a SEM framework for categorical data analysis in order to handle both dimensionality reduction and regression techniques within the same model (cf. the right part of Figure 1).

Two major recent developments in handling categorical data include Muthen's extension of SEM to the 'latent variable modeling' approach [17] and an extension of generalized linear models to latent and mixed variables under GLLAMM (Generalized Linear Latent And Mixed Models) framework [18]. Despite coming from different statistical backgrounds, both Muthén's Mplus software [19] and GLLAMM are capable of modelling a mixture of continuous, ordinal and nominal scale variables, multiple groups (including clusters) and hierarchical (multi-level) data, random effects, missing data, latent variables (including latent classes and latent growth models) and discrete-time survival models. Both of these developments are based on the vision of generalized linear models as a unifying framework for both continuous and categorical variables, where the latter are first transformed into continuous linear functions and subsequently modelled by SEM. This paper follows the same line but proposes a different transformation for categorical variables, so far unused in SEM. A simulated and a real data example with a latent confounding variable are presented.

## Methods

### Data generation and transformation

This work illustrates the application of SEM for binary variables using Yule's transformation to approximate the matrix of Pearson's correlation coefficients from odds ratio (OR) by a well known formula (OR-1)/(OR+1). The first example is based on known data generating processes to avoid uncertainty about true model, virtually inevitable for empirical data. A data set with 5000 observations was generated to allow normal theory approximation. First, three continuous random variables, denominated *x*_1 _to *x*_3_, were created from the uniform distribution. The variables were uncorrelated in the population. Their binary versions, denominated *BIN*_1 _to *BIN*_3_, were obtained by coding the values above the mean as one versus zero otherwise. Two continuous dependent variables were created by the following equations: *m *= 1.5 *x*_1 _+ 2 *x*_2 _+ *e*_1 _and *y *= 0.5 *x*_2 _- 2.5 *x*_3 _+ 1.3 *m *+ *e*_2_, with *e*_1 _and *e*_2 _being normally distributed random errors (N~0,1), generated from different seeds. The binary versions of the dependent variables, denominated *MBIN *and *YBIN*, were created by applying the logistic regression classification rule, i.e. score 1 if *exp*(*m*)/(1+*exp*(*m*)) and *exp*(*y*)/(1+*exp*(*y*)) exceed 0.5 versus 0 otherwise, where 'exp' stands for 'exponentiation'.

**Figure 2:**
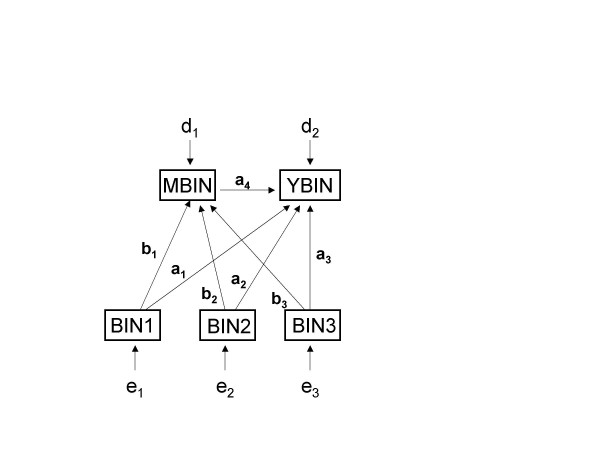
Simulated model.

Observed odds ratios between the variables of interest in the generated data sets are reported in table 1. The structural relationships among the variables in the second data set are depicted in Figure 2.

**Table 1:** Simulated data: Observed odds ratios (OR), associated 95% confidence intervals (CI) and SEM regression coefficients with corresponding standard errors (SE) obtained via ML estimation (N = 5000)

	Observed association		SEM-predicted effects	
Parameter*	OR (95% CI) for the variable pairs	Correlation (Q) estimate	Regression estimate (SE) in Q-metric	Regression estimate (95% CI) in OR-metric**

a_1 _(BIN1→YBIN)	2.138 (1.887, 2.423)	0.3627	0.0281 (0.0039)	1.058 (1.042, 1.074)
a_2 _(BIN2→YBIN)	3.711 (3.255, 4.232)	0.5755	0.1036 (0.0044)	1.231 (1.210, 1.253)
a_3 _(BIN3→YBIN)	0.364 (0.321, 0.414)	-0.4660	-0.4979 (0.0033)	0.335 (0.329, 0.341)
a_4 _(MBIN→YBIN)	10.883 (9.411, 12.586)	0.8137	0.7760 (0.0050)	7.929 (7.554, 8.337)
b_1 _(BIN1→MBIN)	2.632 (2.304, 3.006)	0.4493	0.4479 (0.0093)	2.622 (2.507, 2.746)
b_2 _(BIN2→MBIN)	4.095 (3.561, 4.709)	0.6075	0.6070 (0.0093)	4.089 (3.863, 4.337)
b_3 _(BIN3→MBIN)	1.083 (0.955, 1.229)	0.0398	0.0276 (0.0093)	1.0568 (1.019, 1.096)

* Arrows point to the dependent variables in the model (see Figure 2)

** Back-transformed from Q to OR by (1+Q)/(1-Q)

In addition, a random sample of 100 observations was taken from the generated data set with 5000 observations in order to illustrate small sample performance of the SEM based on Yule's transformation compared to logistic regression. Finally, a real data example with related binary obstetric outcomes, including premature birth, lower segment Caesarian section, low birthweight (<2500 g) and utilization of special baby care unit, was used to compare the SEM with logistic regression as a standard technique applied to this type of data. The data were extracted from obstetric records of 10574 multiparous women with singleton pregnancies who delivered a baby between 1^st ^August 1994 and 31^st ^July 1995 in nine maternity units in England and Wales [20].

Yule's transformation was used to estimate the matrix of Pearson's correlation coefficients for both simulated and real obstetric data. The correlations were used as input for SEM. For the simulated data, both logistic and SEM analysis were repeated for a random subset of 100 observations taken from the original data set. Maximum likelihood (ML) estimation was used.

SEM raw regression coefficients were back-transformed from Q-metric into odds metric by (1+Q)/(1-Q) to get an impact measure for the binary predictor variables. SAS software procedures CALIS and LOGISTIC were used for SEM and logistic analysis, respectively [21].

### Evaluation of classification performance

Raw data residuals were calculated as the difference between observed and SEM-predicted values for both data sets. The predicted values were calculated by multiplying the raw regression parameters obtained in SEM with corresponding observed values of the predictor variables. The back-transformation from SEM parameters, denoted S, to the odds metric is given by (1+S)/(1-S) and provides the odds of being the case for each independent variable; summing these odds over the independent variables gives the odds of being the case for each profile of independent variables. The odds greater than one were classified as SEM predicted cases versus otherwise.

For logistic regression, the percent of correctly classified outcomes was calculated using the cut-off point of 0.5 for the estimated probability of outcome variables.

The classification performance of SEM and logistic regression was compared on a real data set with several obstetric outcomes of interest [20] and on a small random sample of 100 observations taken from the simulated data set of 5000 observations.

### Power analysis

Statistical power analysis used a calculation based on non-central chi-squared distribution, providing the number of observations required to achieve the 90% power (beta or type II error of 0.10), denoted as *N *[22,23]. If *n *denotes the number of observations used in SEM, *k *denotes the multiplying factor for a chosen power level, degrees of freedom and alpha (type I error), and *d *denotes the chi-square difference between the SEM with and without the parameter(s) of interest, then N = k*n/d gives the required sample size. Releasing one parameter at a time (one degree of freedom), with fixed type I error of 5% and type II error of 10%, point to the tabulated *k*-value of 10.51 [23]. This approach assumes that the model is correctly specified.

## Results

Table 1 contains observed odds ratios for the simulated data set and their decomposition into regression effects based on SEM using Yule's transformation of odds ratios.

A standard approach to the analysis of binary variables using multivariate logistic regression for the simulated data is presented in Table 2.

**Table 2:** Multivariate logistic regression for generated data: parameter estimates (standard errors) for large (N = 5000) and small (N = 100) samples

	YBIN outcome		MBIN outcome	
	N = 5000	N = 100	N = 5000	N = 100

Intercept	-0.5735 (0.0787)	-0.3470 (0.6320)	-0.0835 (0.0627)	0.9316 (0.4745)
BIN1	0.5602 (0.0781)	0.3234 (0.5362)	1.0596 (0.0713)	0.9921 (0.9921)
BIN2	0.9941 (0.0791)	1.3645 (0.5409)	1.4787 (0.0734)	1.4472 (0.5842)
BIN3	-1.5431 (0.0844)	-1.6759 (0.5551)	0.0708 (0.0691)	-0.8168 (0.5530)
MBIN	2.3781 (0.0873)	1.7528 (0.6260)	-	-

**Figure 3:**
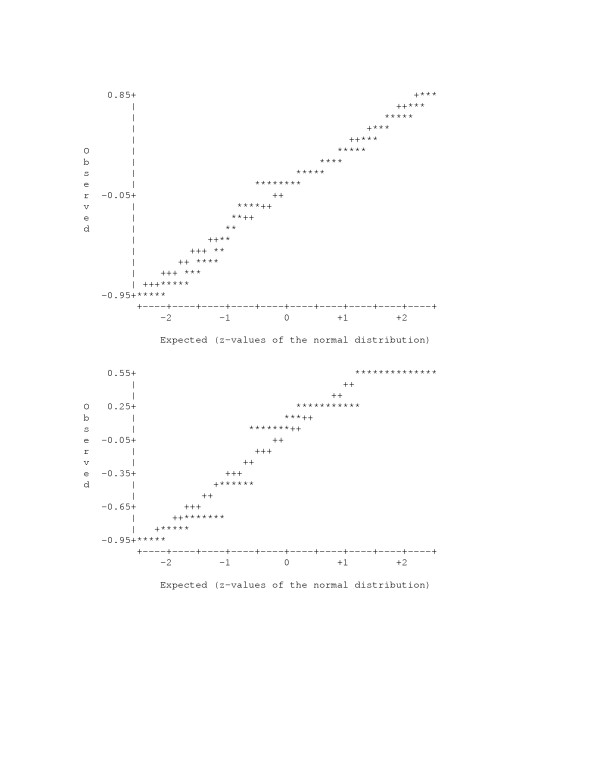
**Normal probability plots for raw data residuals**. Normal probability plots for raw data residuals in the simulated data model with two related outcomes: YBIN (top) and MBIN (bottom). Asterisk may represent up to 30 residuals.

**Figure 4:**
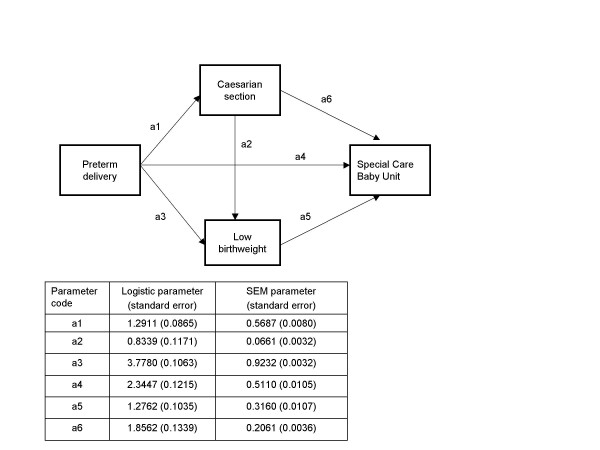
Comparison of SEM and logistic model estimates for the obstetric data example.

The normal probability plot of raw data residuals between observed outcomes and the estimated probability of outcome based on SEM for simulated data showed some departure from the normal distribution (Figure 3). On the other side, the residuals fall within the normal range. Both SEM and logistic regression models for real obstetric data (Figure 4) showed satisfactory fit regarding individual data residuals.

The comparison of classification performance for SEM versus logistic regression showed slightly better results with the latter for one outcome in a small sample analysis and very similar results for all other comparisons (Table 4). True positive fraction for events was always considerably higher for SEM compared to logistic regression, albeit at the expense of lower true negative fraction for non-events.

**Table 3:** Small sample (N = 100) parameter estimates and their standard errors (SE) for SEM using Q-statistic input (correlations estimated via Yule's transformation)

	Observed association		SEM-predicted effects	
Parameter*	OR (95% CI) for the variable pairs	Correlation (Q) estimate	Regression estimate (SE) in Q-metric	Regression estimate (95% CI) in OR-metric**

a_1 _(BIN1→YBIN)	1.600 (0.669, 3.824)	0.2308	0.0068 (0.0410)	1.014 (0.863, 1.191)
a_2 _(BIN2→YBIN)	3.881 (1.561, 9.650)	0.5902	0.4354 (0.0485)	2.542 (2.032, 3.259)
a_3 _(BIN3→YBIN)	0.233 (0.092, 0.594)	-0.6220	-0.5604 (0.0403)	0.282 0.220, 0.350)
a_4 _(MBIN→YBIN)	8.037 (2.700, 23.925)	0.7787	0.3387 (0.0568)	2.173 (1.589, 2.636)
b_1 _(BIN1→MBIN)	2.581 (0.856, 7.782)	0.4415	0.3697 (0.0625)	2.173 (1.657, 2.939)
b_2 _(BIN2→MBIN)	3.857 (1.278, 11.638)	0.5882	0.5854 (0.0625)	3.8239 (2.724, 5.847)
b_3 _(BIN3→MBIN)	0.512 (0.185, 1.418)	-0.3230	-0.3441 (0.0624)	0.4880 (0.364, 0.637)

* Arrows point to the dependent variables in the model (see Figure 2)

** Back-transformed from Q to OR by (1+Q)/(1-Q)

**Table 4:** Percentage of correctly classified events for logistic regression (LR) models in table 2 versus SEM in tables 1 and 3

Sample size	N = 5000				N = 100			
Outcome	YBIN		MBIN		YBIN		MBIN	

Method	LR	SEM	LR	SEM	LR	SEM	LR	SEM

All outcomes	80.36	80.28	74.32	74.36	81.00	80.00	80.00	72.00
Events	91.30	93.32	83.46	91.66	91.18	85.29	80.00	76.25
Non-events	54.42	49.36	48.47	25.42	59.38	68.75	0.00	55.00

Logistic regression showed better overall classification rate due to better prediction of non-events (Table 5). On the other hand, events were better predicted by SEM.

**Table 5:** Classification performance for the obstetric data example (N = 10574): logistic regression (LR) and SEM with Q-metric input (see Figure 4)

Correctly classified (%)	Dependent variables					
	
	Caesarian section		Low birthweight		Special Care Baby Unit	
	LR *	SEM	LR *	SEM	LR *	SEM

All outcomes	85.7	83.8	95.4	83.9	95.3	82.7
Events	0.0	15.5	26.7	72.7	36.5	69.0
Non events	100.0	95.2	99.1	84.6	99.2	83.6

* Note: Logistic regression used the same outcome and predictor variables as the SEM model in Figure 4 but needed separate logistic models for each dependent variable.

**Figure 5:**
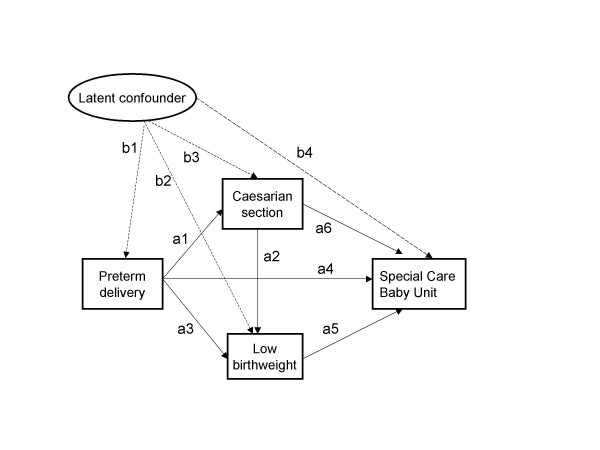
SEM with latent risk variable for the obstetric data example.

SEM permitted further investigation of the unobserved determinant of observed obstetric risk factors in predicting the need for specialised neonatal care through a latent variable. A model was tested assuming that a common cause of some of the risk factors is a latent confounding variable influencing both observed risk factors and the outcome of interest (special baby care unit) and adding predictive power over and above the observed risk variables (Figure 5). The estimation was possible upon solving the observed variables' parameters first (so-called path analysis) and fixing the factor loading for preterm delivery to the value of one – a convention allowing the comparison of the contribution of the other two observed risk variables to the unobserved latent risk using premature birth as unit risk. The factor loadings (standard errors) for Caesarian section and low birthweight were -0.3948 (0.003) and 0.8630 (0.001), respectively.

The relevance of the latent variable for predicting the use of special care baby unit was also tested by linear regression with raw data SEM residuals (observed minus SEM predicted probability of using special care baby unit) as the dependent variable and the latent variable scores as the predictor variable. The predictor was estimated at 0.0874 (standard error 0.0053) and was highly significant (p < 0.001).

The model suggested that propensity for premature birth resulting in low birthweight upon delivery which did not use Caesarian section increased the chances of special neonatal care utilization. The raw SEM coefficient representing this effect, denominated *b*_4 _on Figure 5, was estimated at 0.0956 with corresponding standard error of 0.016, leading to a highly significant t-value of 61.54. Transforming back to odds metric via (1+b_4_)/(1-b_4_) resulted in odds ratio of 1.21 and corresponding 95% confidence intervals from 1.14 to 1.29. Although a multivariate logistic regression model for the special baby care unit utilization did not find the above combination of risk factors statistically significant when it was added as interaction term to the risk factors themselves (odds ratio 1.16 with 95% confidence intervals from 0.72 to 1.86), it should be stressed that this is a model different from the above SEM.

Statistical power analysis found that only the *b*_3 _parameter in table 3 would require a larger sample size (N = 5918) than the one available to achieve the 90% power.

## Discussion

The analysis demonstrated the viability of SEM using Yule's Q-transformation of odds ratio as input for binary variables models. On the level of individual data points, the raw data residuals were within the normal range and the discriminant rule for classification of outcomes into events and non-events based on SEM Q-scores performed slightly worse but still similarly to the results based on standard approach using logistic regression. The conclusion holds for the small sample example with generated data and for the real data set tested here. All these elements point out to the feasibility and utility of SEM using Yule's transformation for binary data, principally when complex relationships between the variables are present. For example, the investigation of the common cause of obstetric risk indicators on the outcome of interest identified a latent confounding variable which increased the chances of utilizing special neonatal care over and above the impact of the same risk indicators taken as independent predictors (Figure 5). The interpretation of the latent variable may lead to hypothesising a health service routine of treating premature births in a particular way (i.e. restraining from Caesarian section) or a biological propensity for birth complications, with both of these alternatives leading to an increased need for intensive neonatal care. This illustrates how SEM helps generating and investigating complex hypothesis not available by other methods. Yule's transformation may be helpful in preparing binary data for SEM. By using odds ratio both as a starting point and for the results presentation, the proposed transformation facilitates the interpretation of effects in the model.

For alpha level <0.05, both the univariate t-test and the likelihood ratio test for the *b*_3 _parameter being equal to zero indicated its statistical significance in SEM (details not shown) despite non-significance of observed odds ratio (table 3). However, the power of this test is less than the pre-established criterion of 90% and the impact of this parameter is clearly inferior to that of the other predictors in the model. The tendency to include extra parameters was also reported for SEM ML estimates where ordered categorical variables were treated as continuous [24] and may be expected for ADF estimates in SEM with raw binary data input. It should be noted that binary variables and the amount of noise introduced in the model analysed are serious obstacles to specifying the correct relationship between the variables for ADF estimation methods, typically applied to the data with smaller departure from the multivariate normal distribution. However, there has been some progress in developing both large sample and finite sample robustness of SEM parameters in handling non-normal data and outliers [25,26].

The advantage of SEM over separate logistic regression models for each outcome is twofold. First, SEM can model all regression equations simultaneously, thus providing a flexible framework for testing a range of possible relationships between the variables in the model, including mediating effects and possible latent confounding variables. Second, on a more general level, SEM parameters can quantify the contribution of each predictor to the covariance structure such as common factors model (Figure 5 is an example), whereas neither the interaction of continuous variables, defined as their crossproduct, nor the interaction terms for categorical independent variables in a regression model, can do this. The modelling of a common cause of observed risk factors and its influence on the outcome of interest is impossible outside SEM framework. Genetic propensity for various diseases is probably the most vivid example of the need for above model, enabling an investigation of the latent confounding variables frequently cited in the study design literature. This includes latent growth models with a relatively long sequence of indicators of an evolving process such as disease whose symptoms are typically binary indicators used for statistical modelling of the outcomes of interest. It is no coincidence that some recent developments in regression modelling have been marked by the efforts to integrate regression with a variety of covariance structure models [1-3].

Another advantage of SEM using Yule's Q-transformation of odds ratios for binary variables over two-level approach, based on probit or logit model or estimated correlations for non-normal variables as first level and SEM as second level modeling, may lay in the fact that the former is based on data transformation rather than estimation, thus avoiding the sources of error due to the latter. However, this view is not universally accepted and the discussion goes back to the beginning of the 20^th ^century when Karl Pearson and George Udney Yule argued whether a measure of association of two binary variables needs to assume underlying continuum and bivariate normal distribution [16]. While the former based his calculation of tetrachoric correlation on these assumptions, the latter disagreed, saying that some categorical variables are inherently discrete, so that the continuum assumption is tenuous and in fact unnecessary because a measure of association for such cases can be obtained directly from cell counts in a 2 by 2 table as in odds ratio and its transformation, today known as Yule's Q. Although the popularity of odds ratio over Pearson's correlation in medical statistic points to a prevailing tendency of embracing Yule's view in this field, an attempt to reconcile the two viewpoints has been made [16].

The fact that Yule's transformation is well known and allows an easy back-transformation of model parameters to odds metric makes it easier to interpret them as effect measures. Although SEM estimates based on already existing methods for handling categorical variables could be converted to an odds ratio metric for the purpose of interpretation, it has been used very rarely in the publications in the field and almost exclusively with GLLAMM.

Usual tools for evaluating SEM fit such as the analysis of residuals are available not only for input covariance matrix but also for individual data points. When classification of outcomes into events and non-events is of interest, sensitivity and specificity parameters can easily be obtained, thus making this approach applicable to a wide range of research problems.

Although other measures of comparative model fit, abundant in the SEM literature [9], may also be useful to assess various aspects of this important issue, classification performance is a preferred measure of predictive power in practice, particularly if cross-validated. For example, both data sets analysed here used saturated models which perfectly predicted the input correlation matrices, so the fit indices based on the discrepancy between observed and SEM-predicted correlation matrices obtained maximum values possible, but this was not particularly informative. On the other hand, SEM fit indices may be useful to select the best model in many other situations.

Despite the advantages of SEM mentioned above, there are several limitations of this work. First, Yule's Q is not exactly Pearson's correlation coefficient but rather an approximation to it which seems reasonable in large samples and for the types of models tested. Although the illustration of a small sample size performance seems satisfactory compared to logistic regression models, it is yet to be tested fully for a much wider range of dependency structures than presented here in order to evaluate the robustness of the parameters obtained. However, this requirement is a consequence of complex modelling issues which often arise in SEM as Yule's Q is no new estimator. Therefore, the findings about the properties of ML, ADF and least squares estimators in SEM, accumulated for almost three decades of research, apply here. This is the main reason why no attempt of a simulation study of SEM parameter estimates has been made in this work. Second, the lack of a simple rule for variable selection in SEM and the need to test a variety of models before selecting the acceptable ones can make it difficult to use this approach for quick decision making often favoured in routine applications of medical statistics. Model selection based on Bayes factors [27] may be helpful in this situation. Finally, although logit is the most popular transformation in modelling binary outcomes in medical statistics, there are many other link functions which may be more suitable for a particular model. GLLAMM [18] theory and software seem to be the most complete framework for such investigation up to date.

When the scale of SEM variables is not equal or their variances differ significantly, covariance matrix input should be preferred instead of correlation matrix input. Although SEM standard errors are less accurate with the latter even with the sample size of few hundreds, the data used here had much larger sample sizes and therefore are less influenced by the type of input matrix. In addition, the input of all SEM variables was on the same scale, i.e. in the odds metric. On the other hand, many SEM applications are performed on moderate and small samples, so the covariance matrix input would be preferable. With multivariate normal distribution, sample covariance matrix contains all the necessary information for SEM. However, with non-normal data, kurtosis was shown to be the most relevant parameter to be taken into account to correct the standard errors of SEM parameters, as in ADF estimators [12]. If means are of interest in SEM, input covariance matrix can be augmented with this information as well. Another way of dealing with SEM standard errors from non-normal data is bootstrapping, already included in several statistical packages with SEM module.

If the raw regression parameters from SEM exceed the domain of the inverse of Yule's transformation function, i.e. the interval from -1 to 1, then standardized SEM parameters can be used to get the odds metric via (1+Q)/(1-Q). Alternatively, a transformation mapping the raw SEM coefficients to this interval may be used, such as Yule's or logit, with corresponding back-transformation of the results to odds metric.

Although this work does not address the question of the association between continuous and dichotomous variables, extensions to include this case can be envisaged. One strategy would be to transform continuous variables into ordered categories with one of them serving as a baseline and then calculate odds ratios using logistic regression. Subsequently, Yule's transformation can be used to convert the odds into correlation metric to be analyzed by SEM. Another strategy would be to use polychoric or poliserial correlation for above situation and only substitute tetrachoric correlation by Yule's Q, particularly when the structural relationships of interest are between binary variables in the model and some exogenous variables are ordered or continuous.

Further research is needed to elucidate various aspects of the SEM based on Q-metric input, particularly small sample performance for a wide range of statistical models and their classification performance. In addition, the variance of odds ratios may be used to weight the estimated correlation matrix, so that Q-metric input for SEM takes into account the precision of the original scale and not only the magnitude of association between two binary variables. Relative fit measures such as those recently proposed by Agresti & Caffo [28] may help selecting among competing models of different kind.

## Conclusion

SEM based on Q-transformation of odds ratios can be used to investigate complex dependency structures such as latent confounding factors and their influences on both observed risk factors and categorical outcome variables.

## Competing interests

The author(s) declare that they have no competing interest.

## Pre-publication history

The pre-publication history for this paper can be accessed here:


